# Effects of Probucol on Restenosis after Percutaneous Coronary Intervention: A Systematic Review and Meta-Analysis

**DOI:** 10.1371/journal.pone.0124021

**Published:** 2015-04-21

**Authors:** Jichen Liu, Menghao Li, Hao Lu, Weiguang Qiao, Dan Xi, TianTian Luo, Haowei Xiong, Zhigang Guo

**Affiliations:** 1 Division of Cardiology, Nanfang Hospital, Southern Medical University, Guangzhou, 510515, Guangdong, P.R. China; 2 Department of Gastroenterology, Nanfang Hospital, Southern Medical University, Guangzhou, 510515, Guangdong, P.R. China; Harefield Hospital, UNITED KINGDOM

## Abstract

**Background:**

Restenosis after percutaneous coronary intervention (PCI) is a remained clinical problem which limits long-term success of PCI. Although there was recognition that probucol in treating restenosis after percutaneous transluminal coronary angioplasty, the efficacy of probucol on restenosis after stent-implantation is controversial. So this meta-analysis was conducted to investigate the association between probucol and late restenosis.

**Methods:**

Articles were assessed by four trained investigators, with divergences resolved by consensus. PubMed, EMBASE, ScienceDirect and the Cochrane Central Register of clinical trials were searched for pertinent studies. Inclusion criteria were random allocated to treatment and a comparison of probucol-treated patients and control patients (not treated with lipid-lowering drug) undergoing PCI.

**Results:**

Fifteen studies with 859 subjects were analyzed. Major outcome, binary angiographic restenosis defined as >50% stenosis upon follow-up angiography, was significantly decreased with probucol treatment (RR = 0.59 [0.43, 0.80] among vessels, P = 0.0007; and RR = 0.52 [0.40, 0.68] among patients, P<0.00001). Probucol also increased the minimal luminal diameter (SMD = 0.45 [0.30, 0.61], P<0.00001) and decreased late loss upon follow-up after 6 months (SMD = -0.41 [-0.60, -0.22], P<0.0001). Moreover, there was a significantly lower incidence of major adverse cardiac events (MACE) in the probucol group than control group (RR = 0.69 [0.51, 0.93], P = 0.01).

**Conclusion:**

Probucol is more than a lipid-lowering drug. It is also effective in reducing the risk of restenosis and incidence of MACE after PCI.

## Introduction

Coronary heart disease (CHD) is the leading cause of death and disability worldwide. Percutaneous coronary intervention (PCI) with stent placement is the standard nonsurgical treatment for CHD. However, restenosis after PCI remains an important clinical problem that occurs in patients who have undergone either percutaneous transluminal coronary angioplasty (PTCA) (30%-50%)[1, 2] or stent-implantation (15%-20%)[3, 4].

Post angioplasty restenosis is thought to involve vessel elastic recoil, negative remodeling[5], smooth muscle cell migration and proliferation[6] and excessive extracellular matrix production[7]. Success of stenting contributes to reducing acute elastic recoil and long-term vessel remodeling[8]. Smooth muscle cell migration and proliferation and excessive extracellular matrix production appears to be main causes of post PCI restenosis.

Probucol has demonstrated its ability in inhibiting vascular smooth muscle cell proliferation after balloon injury in different animal models[9, 10]^.^ In some clinical trials, probucol was effective in reducing restenosis after percutaneous balloon angioplasty. However, its role in the prevention of restenosis after stenting has not been demonstrated in human. Moreover, results have been contradictory and controversial in clinical trials. These inconsistent results may be clarified via a meta-analysis of randomized controlled trials. Thus, we designed a meta-analysis to assess whether treatment with probucol reduced diameter restenosis in patients after PCI.

## Methods

### Search strategy and selection criteria

This meta-analysis was performed in accordance with the PRISMA (Preferred Reporting Items for Systemic Reviews and Meta-Analyses) recommendations[11]. Electronic databases (PubMed, EMBASE, ScienceDirect and the Cochrane Central Register of clinical trials) were searched using the following subject terms: “coronary artery disease”, “cardiovascular disease”, “percutaneous coronary intervention”, “percutaneous transluminal coronary angioplasty”, “stent”, “probucol”, “restenosis”, “minimal luminal diameter” and “late loss”, through January 30, 2015. The search was limited to randomized controlled trials and human studies without language restrictions. We also hand-searched the reference lists of studies, including reviews of probucol and other types of articles related to our primary subject matter, to ensure other relevant articles.

Only randomized controlled trials (RCTs) comparing probucol with control treatments (placebo, standard care without any lipid-lowering drugs) for patients with coronary atherosclerosis disease who underwent PCI were included. Follow-up coronary arteriography was conducted 6 months later to evaluate lumen diameter of PCI segments. Our study’s major outcome was binary angiographic restenosis defined as a diameter stenosis>50% at follow-up. Other studies containing minimal diameter (MLD) or late loss were also our interests. The second outcome was incidence of major adverse cardiac events (MACE), including death, myocardial infarction (MI), repeated angioplasty and coronary artery bypass surgery (CABG). If several groups were included in a single study, only the probucol group and the control group were included in our meta-analysis.

### Data collection and study quality

Three researchers (JC Liu, MH Li, H Lu) independently reviewed references and abstracts retrieved by the search, assessed the completeness of the data abstraction and confirmed quality rating. The quality of each study was independently evaluated by using the Risk of Bias Table from the Cochrane Collaboration. Each factor was rated as ‘‘low risk” of bias (e.g., random sequence generation was computer generated), ‘‘high risk” of bias (e.g., participants and personnel were not blinded) or ‘‘unclear risk” of bias (e.g., methods used for allocation concealment were not described in the manuscript). We redefined low risk, high risk and unclear risk of bias in incomplete outcome data as less than 20%, more than 40% and 20–40% censored data. Any disagreement regarding data collection or the quality assessment was adjudicated by the fourth reviewer (ZG Guo).

### Statistical analyses

The meta-analysis was conducted by combining the risk ratio of individual studies into a pooled risk ratio for dichotomous outcomes. And the continuous outcomes were analyzed by standard mean difference. Overall estimated effects were calculated with either fix-effects model or with a random-effects model when heterogeneity could not be explained[12]. Heterogeneity[13, 14] among studies was evaluated by the Cochran Q test (considered significant if P<0.1), the Chi-squared test, the *I* ^*2*^ test and tau^2^. Funnel plots were generated for subjectively assessment of publication bias[15].

To verify the consistency of results, the influence of each individual study on the summary effect estimate was assessed by the 1-study removed sensitivity analysis. Subgroup analyses of MLD were conducted according to ethnicity (Asian vs non-Asian), probucol dosage (>500 mg daily vs ≤500 mg daily), stent-implantation (stent vs non-stent) and the duration of drug use before PCI (≥30days vs ≤14days) to explore the source of heterogeneity.

Analyses were performed with RevMan 5.2 software (Review Manager (RevMan) [Computer program]. Copenhagen: The Nordic Cochrane Centre, The Cochrane Collaboration, 2012.)

## Results

### Selected studies and characteristics

**Fig 1:**
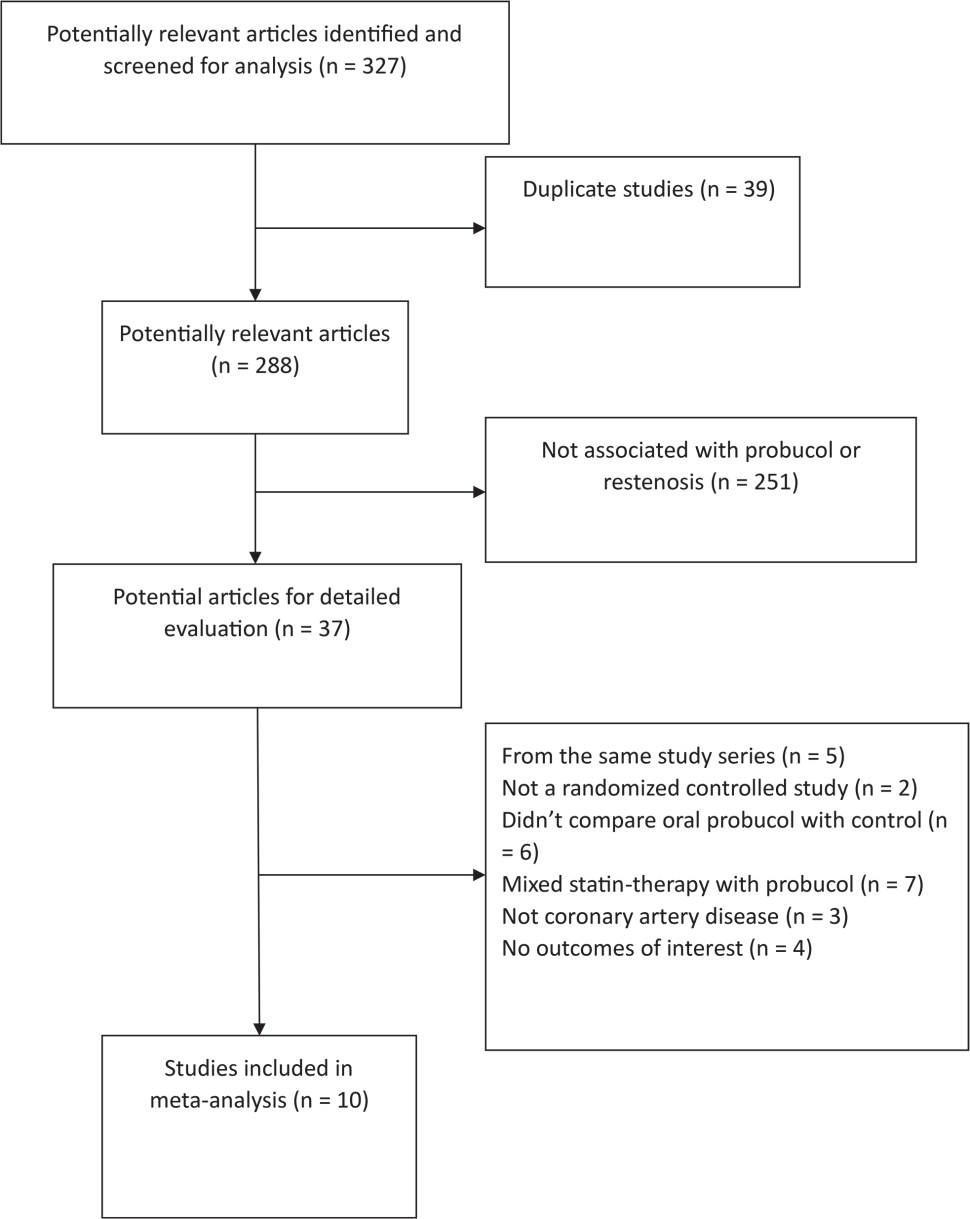
Flowchart of articles included in the meta-analysis.

**Fig 2:**
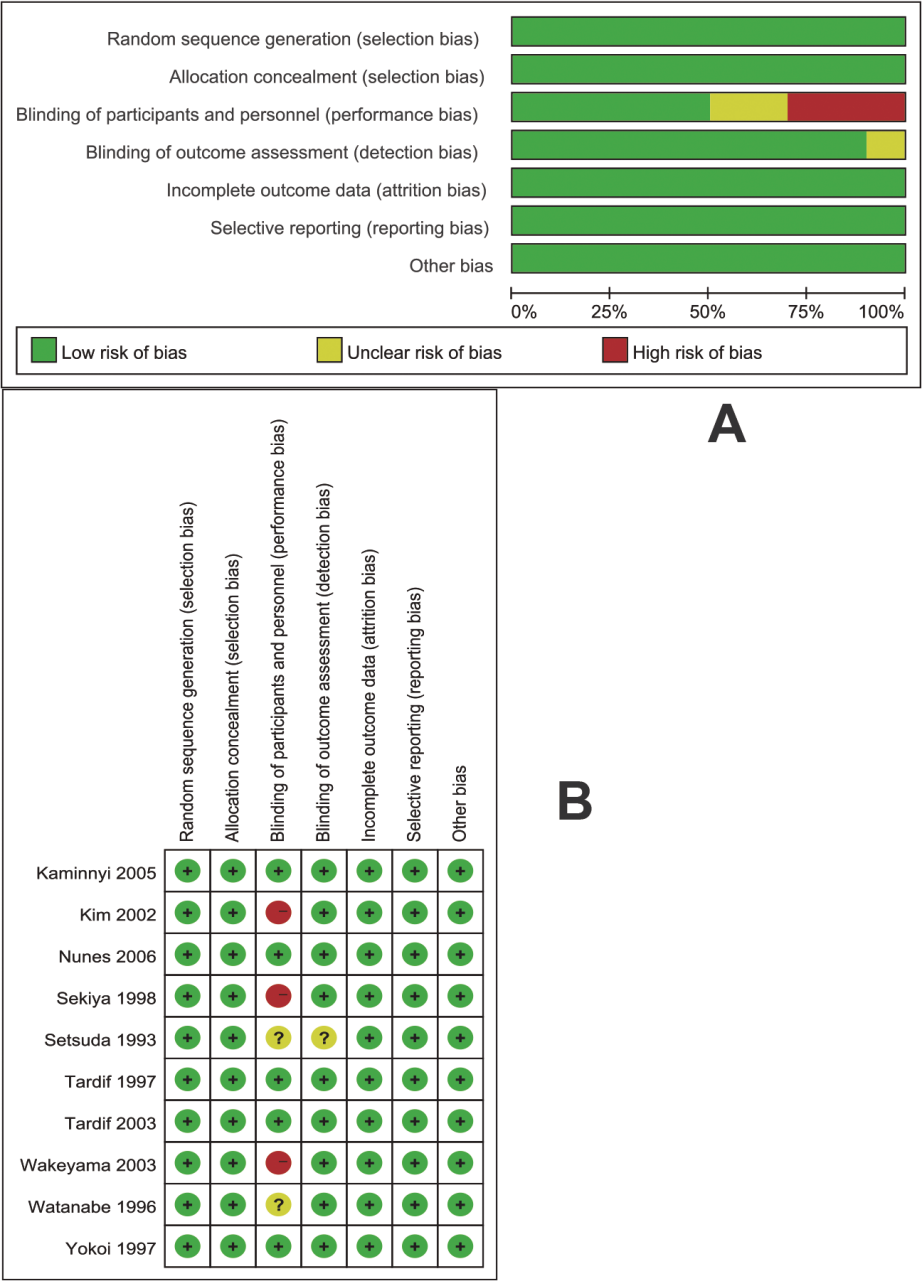
Risk of bias. (A) Risk of bias graph. (B) Risk of bias summary.

The process for the selection of studies for inclusion in the meta-analysis is shown in Fig 1. Our search strategy generated 327 results, 39 of which represented duplicate studies were removed. 37 articles were examined in detail. Of these 37 studies, 27 studies were subsequently excluded: 5 trials were from same study series; 2 trials were not randomized controlled studies; 6 trials did not compare oral probucol with conventional treatments; 7 trials were mixed statin-therapy with probucol-therapy, 3 trials included patients who did not suffer coronary artery disease, and 4 trials contained no outcomes of interest. 10 studies[16–25] were included in the analyses which examined a total of 859 patients. The characteristics of each study are listed in S1 Table. The risk of bias assessment is displayed in Fig 2. Three unblinded studies were determined to be high risk (Kim 2002, Sekiya 1998 and Wakeyama 2003).

### Primary outcome

**Fig 3:**
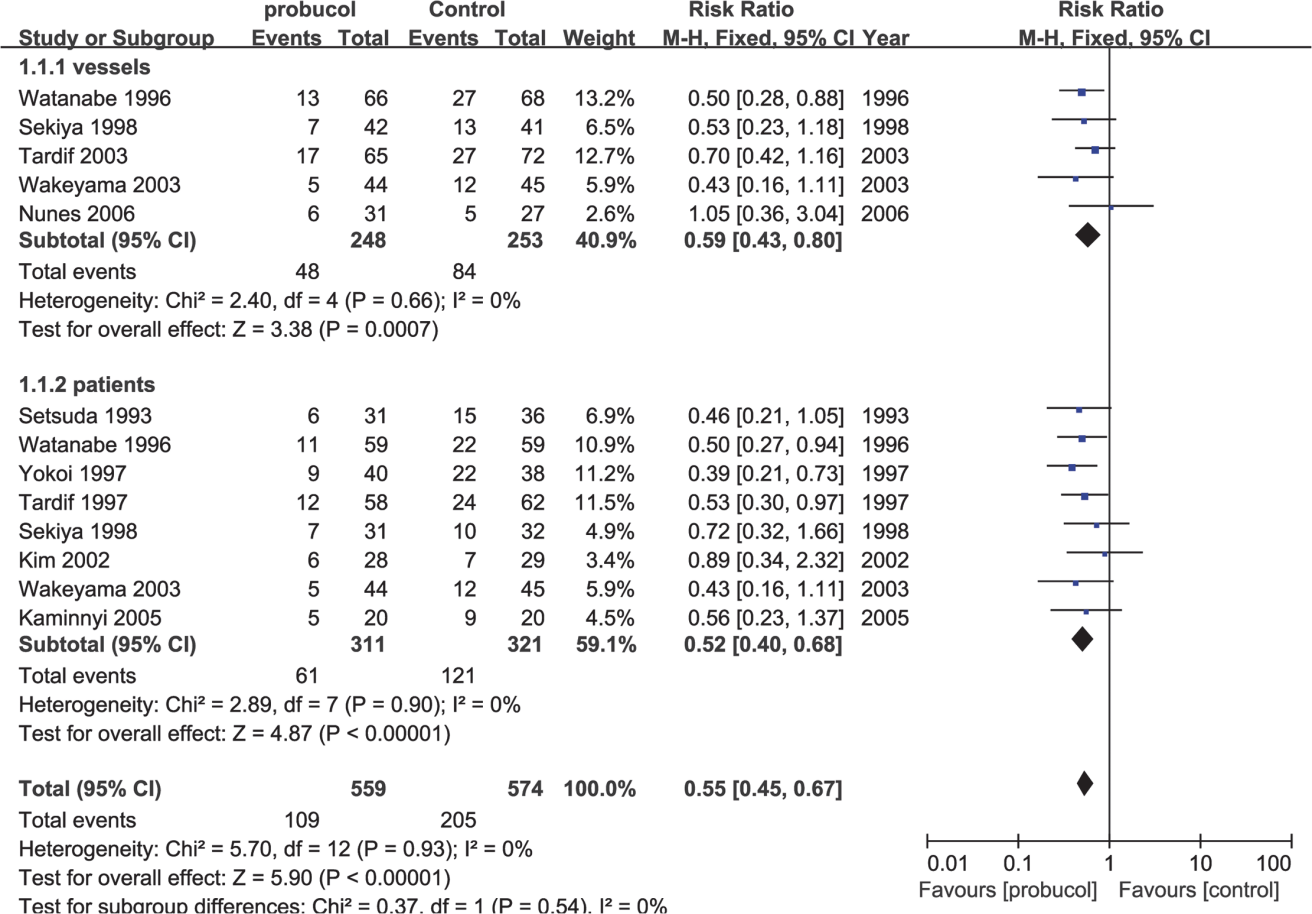
Pooled outcomes of restenosis among PCI segments and patients.

**Fig 4:**
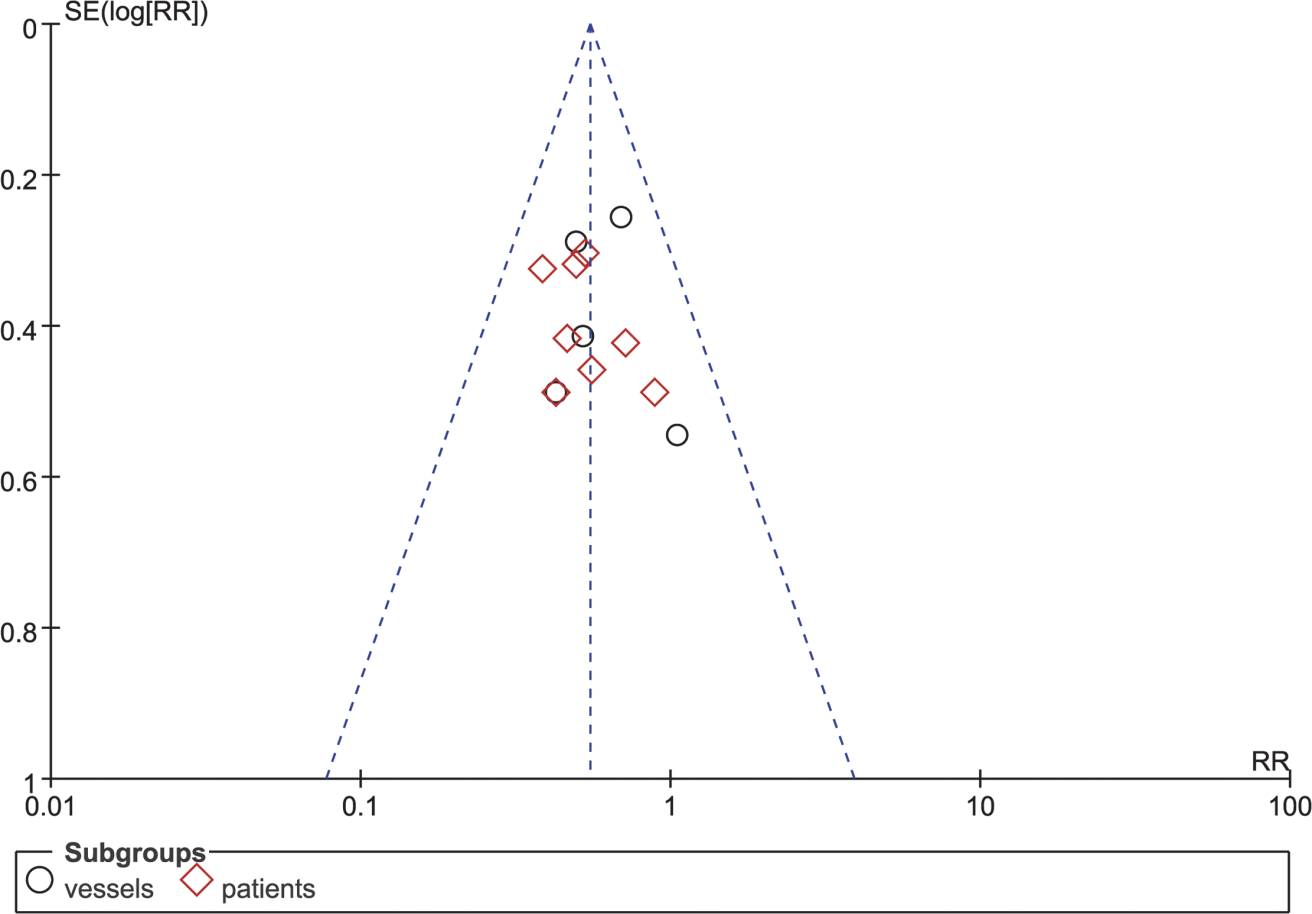
Funnel plot of restenosis.

**Fig 5:**
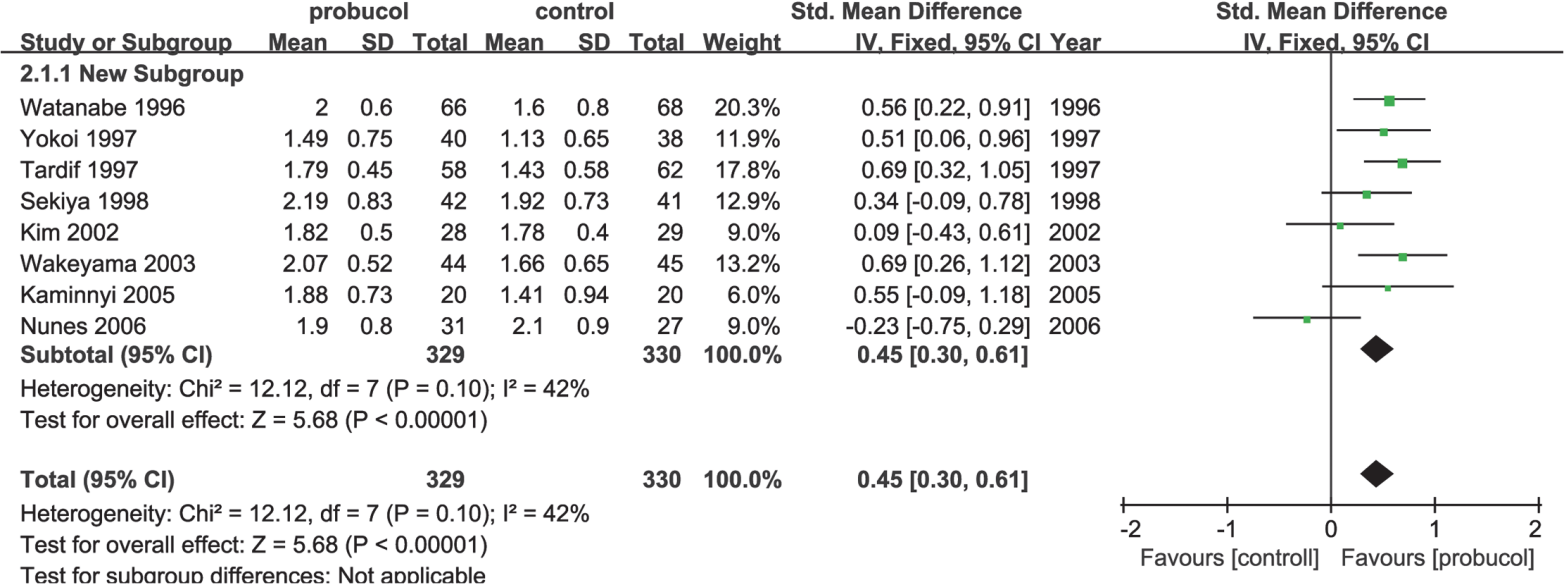
Pooled outcomes of MLD.

**Fig 6:**
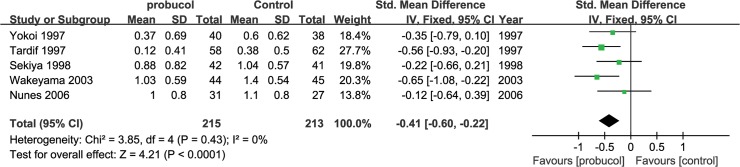
Luminal late loss upon 6-month follow-up.

Our primary outcome was percentage of restenosis, which was measured in 10 studies. The primary outcome was measured in following two ways: the occurrence of restenosis in the PCI segments and the occurrence of restenosis among patients. Probucol reduced the risk ratio of restenosis among both lesions (RR: 0.59 [0.43, 0.80], P = 0.0007, I^2^ = 0) and among patients (RR: 0.52 [0.40, 0.68], P<0.00001, I^2^ = 0) (Fig 3) using a fix-effects model. Probucol nonetheless reduced the frequency of restenosis among patients who underwent stent implantation (RR = 0.66 [0.50, 0.88], P = 0.004) as much as it did among patients who underwent PTCA (RR = 0.47 [0.34, 0.65], P<0.00001). No evidence of publication bias was identified based on funnel plots (Fig 4). An increase in MLD was observed with probucol compared with the control group (SMD = 0.45 [0.30, 0.61], P<0.00001, I^2^ = 42%) (Fig 5), whereas a significant reduction in luminal late loss is shown in Fig 6 (SMD = -0.41 [-0.60, -0.22], P<0.0001, I^2^ = 0%).

### Secondary outcomes

**Fig 7:**
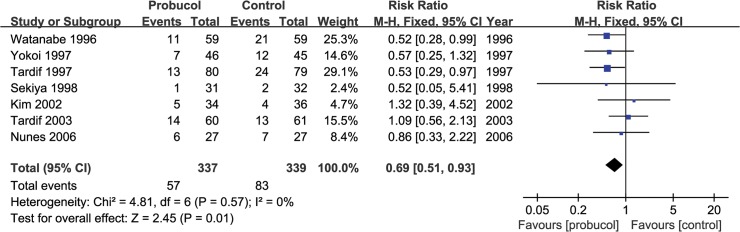
Pooled outcomes of clinical outcomes (death, myocardial infarction, repeat revascularization, coronary artery bypass grafting).

A total of 676 patients in 7 studies were considered in the analysis of MACE. 57 patients in probucol group and 87 patients in control group suffered from cardiac events (death, MI, repeat revascularization and CABG). Treatment of probucol reduced incidence of MACE by 31 percent compared with standard treatment after PCI (RR = 0.69 [0.51, 0.93], P = 0.01, I^2^ = 0%) (Fig 7).

### Subgroup analyses and sensitivity analysis

A sensitivity analysis was conducted to determine the consistency of each outcome. Three high-risk studies were included in the sensitivity analysis. Adding these studies to the pooling only minimally altered the pooled MLD from 0.52 [0.36, 0.68] to 0.40 [0.23, 0.57]. Subgroup analyses of MLD based on ethnicity, dosage and duration of drug use before PCI appeared to have no effect on the results in each group (S2 Table). However, the effect of probucol on MLD among patients who underwent PTCA appeared to be more significant upon follow-up at 6months than the effect among patients who instead underwent stent implantation. (SMD = 0.59 [0.38, 0.80] vs 0.28 [0.04, 0.51], P = 0.05).

## Discussion

This meta-analysis showed that probucol, which has been available for many years, may be useful in a new context. Although the sizes of the populations in many of the trials examined in our analysis were small and the results were conflicting, our meta-analysis nonetheless showed that probucol reduced the risk of restenosis by nearly 50%. Moreover, the incidence of MACE was reduced after probucol treatment without heterogeneity.

Regarding the heterogeneity in MLD that was observed across studies, probucol appeared to be less effective in patients who underwent stent implantation than in patients who underwent balloon angioplasty. This result was most likely due to the difference in the physiopathology of the restenosis that occurred after balloon angioplasty compared with stent replacement. Many studies have demonstrated that restenosis after balloon angioplasty results from early elastic recoil, negative remodeling and neointimal hyperplasia[26]. And the stent virtually eliminated early elastic recoil and negative remodeling. However, the incidence of neointimal hyperplasia increased with balloon angioplasty[27]. Because of these differences, the anti-restenotic effects of probucol on patients who have undergone either PTCA or stent should not be expected to be identical. A meta-analysis of 11 randomized studies found that significantly fewer restenosis occurred following stenting than following PTCA (25.8% vs34.2%)[28]. Stent-implantation reduced restenosis and increase MLD to a larger extent than has been noted in patients who undergo PTCA. Thus, adding probucol therapy, as a treatment for patients who underwent stent-implantation, may not be as effective as this treatment in patients who underwent PTCA. Nonetheless, probucol affected the genesis of intimal hyperplasia and reduced restenosis in patients who underwent stent-implantation.

Besides its antioxidant function, other properties of probucol should be noticed. Properties included promoting endothelial cell growth and functional re-endothelialization[29], inducing heme oxygenase-1 (HO-1) mRNA and HO-1 activity in vascular smooth muscle cell (SMC) to inhibit proliferation of SMCs and intimal thickening[30, 31]. Probucol improved endothelium-dependent arterial relaxation[32] and functional re-endothelialization following aortic balloon injury, as measured via extent of re-endothelialization, nitric oxide (NO) production and nitric oxide-mediated vasodilation in animals. In some clinical trials, plasma NO and flow-mediated vasodilation during reactive hyperemia (FMD) were improved[33], as well as carotid artery intima-media thickness (IMT) and the rate of IMT increased[34]. In addition, probucol may also benefit patients with type 2 diabetes. Some study demonstrated that probucol could reduce blood glucose and preserve β-cell function in some animal models of type 2 diabetes[35, 36]. Probucol protects β cells of the pancreas through its strong anti-free radical and antioxidant effects, thereby neutralizing reactive oxygen species and alleviating oxidative stress. A novel microencapsulated formulation of probucol is being developed and may have potential as an anti-diabetic drug.

Although these data suggest that probucol is effective in reducing the rate of restenosis among patients who have undergone PCI, concerns have been raised about its high-density lipoprotein cholesterol-lowering effect and prolongation of QT interval[37]. Recent trials have indicated that probucol’s HDL-C lowering side-effect did not hamper its ability to alleviate atherosclerosis. More and more studies found that functional HDL subfractions shifted to dysfunctional HDL subfractions during ACS[38]. The assessment of functional HDL had become a novel target to investigate the association between HDL and coronary artery disease risk[39]. Previous studies from our group[40] and from other institutions have demonstrated that probucol reduced HDL-C levels but promoted enhanced reverse cholesterol transport (RCT) via activation of CETP and scavenger receptor class B type I (SR-BI)[41, 42]. Moreover, some studies suggested that probucol may have beneficial effect on the plaque stability in vivo[43, 44]. However, QT interval prolongation will limit its long-term use. New probucol analogues or preparations may alleviate this side effect. AGI-1067, the mono succinic acid ester of probucol, is a metabolically stable compound that has greater intracellular antioxidant efficacy in vitro than probucol without its QT-interval prolonging effect[45, 46]. Similarly to probucol, AGI-1067 significantly reduced the in-stent restenosis compared with placebo in the CART-1 study[22]. However, AGI-1067 did not reduce the study’s primary endpoint, i.e. the time to the first occurrence of cardiovascular death, stroke, coronary revascularization in the ARISE study[47]. In addition, a novel polymer-free sirolimus- and probucol-eluting stent demonstrated both its safety on QT-interval prolonging issues and anti-restenotic efficacy in several clinical trials[48, 49]. These new formulations of probucol may represent new directions for its clinical use.

## Limitation

Our meta-analysis included 10 RCTs to acquire as much information as possible. However, our study had several limitations. First, we had no access to individual patient data, including data regarding the underlying diseases from which each patient suffered, the stent types, the specific conventional drugs used in control group, base-line blood lipid values, or reference luminal diameters (RLDs) where either stents or balloons had been used. Each of these issues may have affected the results of our analysis and may have been the cause of the heterogeneity observed in MLD. Some clinical trials suggested that coronary stenting in smaller vessels was associated with an increased risk of subacute thrombosis[50]. Rodés et al demonstrated that probucol reduced both lumen loss and restenosis after balloon angioplasty in small coronary arteries (vessels with RLD<3.0 mm)[51]. Second, our meta-analysis considered a follow-up period of 3–6 months, the period during which restenosis most often occurred^2^. A follow-up period of 6 months seemed to be too short to determine with certainty the effect of probucol on restenosis or MACE after PCI. The PART study extended the observation period to 1 year and found that probucol still facilitated a significant reduction in the incidence of repeat revascularization[52]. Moreover Kasail[53] et al conducted a propensity analysis and found that probucol therapy improved long-term (>10-year) survival after complete revascularization. Despite these findings, more large scale clinical trials are required to clarify the long-term benefits of probucol.

## Conclusion

In conclusion, the available data demonstrated that probucol significantly reduced the restenosis and late luminal loss, and increased MLD upon follow-up at 3-6-month in patients who underwent PCI compared with patients who were not treated with any lipid-lowering drugs. Moreover treatment with probucol reduced the incidence of MACE and probably increased the long-term survival rate after PCI.

## Supporting Information

S1 PRISMA Checklist (DOC)

S1 File List of full-text excluded articles. (DOCX)

S1 Table Study characteristics. Note: M, men; P, probucol group (standard drug treatment plus only probucol); C, control group (usual drug treatment without any lipid-lowering drugs); Duration*: duration of drug-use before PC
(DOCX)

S2 Table Subgroup analyses. (DOCX)
