# Supplementary material for: Effects of Probucol on Restenosis after Percutaneous Coronary Intervention: A Systematic Review and Meta-Analysis
Source: PLoS One. 2015 Apr 21;10(4):e0124021. doi: 10.1371/journal.pone.0124021 (PMC4405356; doi:10.1371/journal.pone.0124021)
Supplement: S1 File — (DOCX) [file pone.0124021.s002.docx]

**List of full-text excluded articles**

There are 37 potential articles we re-evaluate with full text. And 27 articles were excluded for following reasons.

**From the same study series (n = 5)**

1. Cote G, Tardif JC, Lesperance J, et al. Effects of probucol on vascular remodeling after coronary angioplasty. Multivitamins and Protocol Study Group. *Circulation* 1999; 99(1): 30-5.

2. Daida H, Kuwabara Y, Yokoi H, et al. Effect of probucol on repeat revascularization rate after percutaneous transluminal coronary angioplasty (from the Probucol Angioplasty Restenosis Trial [PART]). *Am J Cardiol* 2000; 86(5): 550-2, A9.

3. Tardif JC, Cote G, Lesperance J, et al. Impact of residual plaque burden after balloon angioplasty in the MultiVitamins and Probucol (MVP) trial. *Can J Cardiol* 2001; 17(1): 49-55.

4. Rodes J, Cote G, Lesperance J, et al. Prevention of restenosis after angioplasty in small coronary arteries with probucol. *Circulation* 1998; 97(5): 429-36.

5. Lee HS, Tardif JC, Harel F, et al. Effects of plaque composition on vascular remodelling after angioplasty in the MultiVitamins and Probucol (MVP) trial. *Canadian Journal of Cardiology* 2002; 18(3): 271-5.

**Not a randomized controlled study (n = 2)**

1. Kasai T, Miyauchi K, Kubota N, Kajimoto K, Amano A, Daida H. Probucol therapy improves long-term (>10-year) survival after complete revascularization: a propensity analysis. *Atherosclerosis* 2012; 220(2): 463-9.

2. Schneider JE, Berk BC, Gravanis MB, et al. Probucol decreases neointimal formation in a swine model of coronary artery balloon injury: A possible role for antioxidants in restenosis. *Circulation* 1993; 88(2): 628-37.

**Didn’t compare oral probucol with control (n = 6)**

1. Byrne RA, Mehilli J, Iijima R, et al. A polymer-free dual drug-eluting stent in patients with coronary artery disease: a randomized trial vs. polymer-based drug-eluting stents. *European heart journal* 2009; 30(8): 923-31.

2. Massberg S, Byrne RA, Kastrati A, et al. Polymer-free sirolimus- and probucol-eluting versus new generation zotarolimus-eluting stents in coronary artery disease: the Intracoronary Stenting and Angiographic Results: Test Efficacy of Sirolimus- and Probucol-Eluting versus Zotarolimus-eluting Stents (ISAR-TEST 5) trial. *Circulation* 2011; 124(5): 624-32.

3. Chen SL, Ye F, Zhang JJ, et al. Real polymer-free sirolimus- and probucol-eluting versus biodegradable polymer sirolimus-eluting stents for obstructive coronary artery disease: DKPLUS-Wave 1, a multicenter, randomized, prospective trial. *Cardiovascular Therapeutics* 2013; 31(4): 193-200.

4. Yu M, Xu B, Kandzari DE, et al. First report of a novel polymer-free dual-drug eluting stent in de novo coronary artery disease: Results of the first in human BICARE trial. *Catheterization and Cardiovascular Interventions* 2014; 83(3): 405-11.

5. King L. Two-year follow-up of a polymer-free sirolimus- and probucol-eluting versus a new generation zotarolimus-eluting stents in coronary artery disease. *EuroIntervention* 2012; 8: N17.

6. Navarese EP, Kowalewski M, Cortese B, et al. Short and long-term safety and efficacy of polymer-free vs. durable polymer drug-eluting stents. A comprehensive meta-analysis of randomized trials including 6178 patients. *Atherosclerosis* 2014; 233(1): 224-31.

**Mixed statin-therapy with probucol (n = 7)**

1. O'Keefe JH, Jr., Stone GW, McCallister BD, Jr., et al. Lovastatin plus probucol for prevention of restenosis after percutaneous transluminal coronary angioplasty. *Am J Cardiol* 1996; 77(8): 649-52.

2. Abe Y, Midorikawa H, Muroi S, et al. Multidrug hybrid therapy including an angiotensin receptor antagonist to prevent restenosis after coronary stenting (preliminary report). [Japanese]. *Japanese Journal of Interventional Cardiology*, 2002; 17(3): 259-62.

3. Ko YG, Choi SH, Chol Kang W, Kwon Lee B, Wook Kim S, Shim WH. Effects of combination therapy with cilostazol and probucol versus monotherapy with cilostazol on coronary plaque, lipid and biomarkers: SECURE study, a double-blind randomized controlled clinical trial. *J Atheroscler Thromb* 2014; 21(8): 816-30.

4. Lee YJ, Daida H, Yokoi H, et al. Effectiveness of probucol in preventing restenosis after percutaneous transluminal coronary angioplasty. *Jpn Heart J* 1996; 37(3): 327-32.

5. Herrera AM, Minguez JRL, Ruiz FA, et al. Restenosis rate reduction after coronary angioplasty with simvastatin and probucol. *Revista Espanola de Cardiologia* 1999; 52(10): 778-84.

6. Wang Jingping, An Jian, Zhang Wutang, et al. Probucol and pravastatin in the prevention of restenosis after percutaneous coronary intervention. Chinese Journal of Interventional Cardiology 2009; (05): 251-4.

7. Zeng Zhiheng,Wu Minsheng, Liu Tangwei, et al. The effect of probucol and fu fang dan shen drops alone and in combination on restenosis after intracoronary stenting. Journal of Guangxi Medical University 2004; (06): 809-813.

**Not coronary artery disease (n = 3)**

1. Gallino A, Do DD, Alerci M, et al. Effects of probucol versus aspirin and versus brachytherapy on restenosis after femoropopliteal angioplasty: the PAB randomized multicenter trial. *J Endovasc Ther* 2004; 11(6): 595-604.

2. Wada N, Sekiguchi M, Yoshioka N, et al. Effect of probucol and pravastatin on common-carotid atherosclerosis in diabetic patients with hypercholesterolemia. *Journal of the Japan Diabetes Society* 2005; 48(1): 53-6.

3. J J, Olsson AG, Bergstrand L, et al. Lowering of HDL2b by probucol partly explains the failure of the drug to affect femoral atherosclerosis in subjects with hypercholesterolemia. A Probucol Quantitative Regression Swedish Trial (PQRST) Report. *Arterioscler Thromb Vasc Biol* 1995; 15(8): 1049-56.

**No outcomes of interest (n = 4)**

1. Sawayama Y, Maeda S, Ohnishi H, Okada K, Hayashi J. Effect of probucol on elderly hypercholesterolemic patients in the FAST study. *Fukuoka Igaku Zasshi* 2006; 97(1): 15-24.

2. Tagawa T, Urabe Y, Kimura Y, Suzuki S, Ono H, Takeda K. Long-term treatment with probucol improves endothelial function in patients with coronary artery disease. *Hypertens Res* 2004; 27(5): 311-8.

3. Zheng XY, Liu L, Zhao SP. [Effects of atorvastatin, alone and in combination with probucol on endothelial function in patients with acute coronary syndrome]. *Zhonghua Xin Xue Guan Bing Za Zhi* 2009; 37(10): 900-3.

4. Tardif JC, Gregoire J, L'Allier PL, et al. Effects of the antioxidant succinobucol (AGI-1067) on human atherosclerosis in a randomized clinical trial. *Atherosclerosis* 2008; 197(1): 480-6.
