# Supplementary material for: Effects of Probucol on Restenosis after Percutaneous Coronary Intervention: A Systematic Review and Meta-Analysis
Source: PLoS One. 2015 Apr 21;10(4):e0124021. doi: 10.1371/journal.pone.0124021 (PMC4405356; doi:10.1371/journal.pone.0124021)
Supplement: S1 Table — Note: M, men; P, probucol group (standard drug treatment plus only probucol); C, control group (usual drug treatment without any lipid-lowering drugs); Duration*: duration of drug-use before PC (DOCX) [file pone.0124021.s003.docx]

| **Trial** | **Sample size(M)** | **Age(y)±SD** | | **Dose(mg/d)** | **Country** | **follow-up** | **Stent** | **Duration*** |
| --- | --- | --- | --- | --- | --- | --- | --- | --- |
|  |  | **P** | **C** |  |  |  |  |  |
| **Setsuda 1993^16^** | 67(51) | 58.8±8 | 61±7.9 | 750/1000 | Japan | 3-6 m | No | >7 days |
| **Watanabe 1996^17^** | 118(91) | 62.7±10.1 | 64.1±11.7 | 500 | Japan | 3-6 m | No | 7 days |
| **Tardif 1997^18^** | 159(126) | 58.5±9.3 | 60.3±8.4 | 500 | Canada | 5-7 m | N0 | 30 days |
| **Yokoi 1997^19^** | 78(64) | 60±9 | 60±9 | 1000 | Japan | 24 w | N0 | 4 weeks |
| **Sekiya 1998^20^** | 63(44) | 64±11 | 65±10 | 500 | Japan | 6 m | Yes | 5 days |
| **Kim 2002^21^** | 70(49) | 57.83±7.51 | 55.8±7.89 | 500 | Korea | 6 m | Yes | 3 days |
| **Tardif 2003^22^** | 121(97) | 61.1±10.9 | 58.2±10.1 | 1000 | Canada | 5-7 m | Yes | 14 days |
| **Wakeyama 2003^23^** | 89(66) | 65±8 | 67±9 | 1000 | Japan | 6 m | Yes | No |
| **Kaminnyi 2005^24^** | 40(40) | 55.0±5.4 | 55.0±5.4 | 250 | Russia | 6 m | No | 7-10 days |
| **Nunes 2006^25^** | 54(34) | 59.1±9.1 | 60.1±9.7 | 1000 | Brazil | 6 m | Yes | 14 days |

**Table 1: Study characteristics**

**Note:** M, men; P, probucol group (standard drug treatment plus only probucol); C, control group (usual drug treatment without any lipid-lowering drugs); Duration*: duration of drug-use before PCI
