# Supplementary material for: Effects of Probucol on Restenosis after Percutaneous Coronary Intervention: A Systematic Review and Meta-Analysis
Source: PLoS One. 2015 Apr 21;10(4):e0124021. doi: 10.1371/journal.pone.0124021 (PMC4405356; doi:10.1371/journal.pone.0124021)
Supplement: S2 Table — (DOCX) [file pone.0124021.s004.docx]

| **subgroup analyses** | **standard mean difference (95% CI)** | **P-value between subgroups** |
| --- | --- | --- |
| **Ethnicity** |  |  |
| Asian | 0.47 [0.28, 0.66] | 0.71 |
| Non-Asian | 0.41 [0.14, 0.68] |  |
| **PTCA vs STENT** |  |  |
| PTCA | 0.59 [0.38, 0.80] | 0.05 |
| STENT | 0.28 [0.04, 0.51] |  |
| **Dosage** |  |  |
| >500 mg | 0.38 [0.12, 0.65] | 0.53 |
| ≤500 mg | 0.49 [0.29, 0.68] |  |
| **Duration of drug use before PCI** |  |  |
| ≥30 d | 0.38 [0.20, 0.57] | 0.18 |
| ≤14 d | 0.61 [0.33, 0.90] |  |

**Table 2: Subgroup analyses**
